# The parthenocarpic gene *Pat-k* is generated by a natural mutation of *SlAGL6* affecting fruit development in tomato (*Solanum lycopersicum* L.)

**DOI:** 10.1186/s12870-018-1285-6

**Published:** 2018-04-27

**Authors:** Rihito Takisawa, Tetsuya Nakazaki, Tsukasa Nunome, Hiroyuki Fukuoka, Keiko Kataoka, Hiroki Saito, Tsuyoshi Habu, Akira Kitajima

**Affiliations:** 1 Graduate School of Agriculture, Kyoto University, Kizugawa, 619-0218 Japan; 2 NARO Institute of Vegetable and Floriculture Science, Tsu, 514-2392 Japan; 3 NARO Institute of Vegetable and Tea Science, Tsu, 514-2392 Japan; 4 Graduate School of Agriculture, Ehime University, Matsuyama, 790-8566 Japan; 5 Present Address: Tropical Agriculture Research Front Japan International Research Center Agricultural Sciences, 1091-1, Kawarabaru, Aza Maezato, Ishigaki, Okinawa 907-0002 Japan

**Keywords:** Parthenocarpy, Tomato, *Pat-k*, QTL, Number of seeds, Ovule

## Abstract

**Background:**

Parthenocarpy is a desired trait in tomato because it can overcome problems with fruit setting under unfavorable environmental conditions. A parthenocarpic tomato cultivar, ‘MPK-1’, with a parthenocarpic gene, *Pat-k*, exhibits stable parthenocarpy that produces few seeds. Because ‘MPK-1’ produces few seeds, seedlings are propagated inefficiently via cuttings. It was reported that *Pat-k* is located on chromosome 1. However, the gene had not been isolated and the relationship between the parthenocarpy and low seed set in ‘MPK-1’ remained unclear. In this study, we isolated *Pat-k* to clarify the relationship between parthenocarpy and low seed set in ‘MPK-1’.

**Results:**

Using quantitative trait locus (QTL) analysis for parthenocarpy and seed production, we detected a major QTL for each trait on nearly the same region of the *Pat-k* locus on chromosome 1. To isolate *Pat-k*, we performed fine mapping using an F_4_ population following the cross between a non-parthenocarpic cultivar, ‘Micro-Tom’ and ‘MPK-1’. The results showed that *Pat-k* was located in the 529 kb interval between two markers, where 60 genes exist. By using data from a whole genome re-sequencing and genome sequence analysis of ‘MPK-1’, we could identify that the *SlAGAMOUS-LIKE 6* (*SlAGL6*) gene of ‘MPK-1’ was mutated by a retrotransposon insertion. The transcript level of *SlAGL6* was significantly lower in ovaries of ‘MPK-1’ than a non-parthenocarpic cultivar. From these results, we could conclude that *Pat-k* is *SlAGL6*, and its down-regulation in ‘MPK-1’ causes parthenocarpy and low seed set. In addition, we observed abnormal micropyles only in plants homozygous for the ‘MPK-1’ allele at the *Pat-k*/*SlAGL6* locus. This result suggests that *Pat-k*/*SlAGL6* is also related to ovule formation and that the low seed set in ‘MPK-1’ is likely caused by abnormal ovule formation through down-regulation of *Pat-k*/*SlAGL6*.

**Conclusions:**

*Pat-k* is identical to *SlAGL6*, and its down-regulation causes parthenocarpy and low seed set in ‘MPK-1’. Moreover, down-regulation of *Pat-k*/*SlAGL6* could cause abnormal ovule formation, leading to a reduction in the number of seeds.

**Electronic supplementary material:**

The online version of this article (10.1186/s12870-018-1285-6) contains supplementary material, which is available to authorized users.

## Background

Parthenocarpy is defined as fruit set and growth without fertilization or other stimulation. In tomato, parthenocarpy is a desirable trait that reduces financial and labor costs of fruit set [1]. In addition, it can increase yield under unfavorable conditions: low or high temperatures, low and high humidity, and low light intensity, all of which inhibit fruit set and growth by impeding the reproductive process [2, 3].

Some natural sources for parthenocarpy in tomato include ‘Soressi’ and ‘Montfavet191’ (*pat*), ‘Severianin’ (*pat-2*), ‘RP75/59’ (*pat3/pat4*), ‘IL5–1’ (*pat4.1/pat5.1*), ‘IVT-line1’ (*pat4.2/pat9.1*), and ‘MPK-1’ (*Pat-k*) [1, 4, 5]. Of these genes, only the *pat-2* gene which is located on chromosome 4, has been isolated thus far; it has been determined to encode a zinc-finger homeodomain protein [6].

The parthenocarpic tomato cultivar ‘MPK-1’ exhibits stable parthenocarpy. Hosokawa [7] reported that ‘MPK-1’ was derived from a cross between a non-parthenocarpic cultivar and a variant from a self-fertilized descendant of ‘Severianin’, which exhibits strong parthenocarpy. It is thought that the parthenocarpic trait of ‘MPK-1’ is derived from *pat-2* because ‘Severianin’ is its only parthenocarpic parent [8]. However, recently we found that the parthenocarpy of ‘MPK-1’ is controlled by a novel parthenocarpic gene, *Pat-k* which is located on chromosome 1 [1].

‘MPK-1’ is commercially cultivated in Kyoto Japan, under the name ‘Kyo-temari’. The seedlings of ‘MPK-1’ must be vegetatively propagated from cuttings because seed production is extremely inhibited in ‘MPK-1’ [9]. Takisawa [8] observed that many ovules of ‘MPK-1’ have an abnormal micropyle, which might cause the inhibition of seed formation in ‘MPK-1’. In addition, ‘MPK-1’ has abnormally-fused sepals [1]. One of the natural parthenocarpy mutants, the *pat* mutant, exhibits partial aberrations of the stamens and ovules [10], indicating that *pat* may result from the mutation of a putative gene with homeotic functions. In addition, down-regulation or mutation in some MADS-box genes in tomato (*TM29*, *TAP3*, *TM8*, or *SlAGL11*) not only causes homeotic conversion in flowers but also parthenocarpic fruit development [11–14]. Therefore, it would be reasonable to assume that *Pat-k* not only causes parthenocarpy but also possesses a homeotic function, which might cause the inhibition of seed formation in ‘MPK-1’.

In this study, we performed QTL analysis, fine mapping, and map-based cloning to isolate *Pat-k* and to elucidate the relationship between parthenocarpy and the inhibition of seed formation in ‘MPK-1’. In addition, we also observed ovule structure of plants that were homozygous for ‘Micro-Tom’ or ‘MPK-1’ allele at the *Pat-k* locus to clarify the effect of *Pat-k* on ovule formation.

## Methods

### Plant materials and plant growth condition

We developed an F_2_ population (*n* = 89) for QTL analysis following the cross between a non-parthenocarpic tomato cultivar, ‘Micro-Tom’, (Tomato Growers Supply Company, Florida, USA) and a parthenocarpic cultivar, ‘MPK-1’, following procedures of Takisawa [1]. For the progeny test, an F_3_ population (*n* = 42) was developed from a single F_2_ plant that was heterozygous at the *Pat-k* locus. We sowed seeds of the F_3_ population in April 2015 and grew them in the greenhouse at the Takatsuki Experimental Farm of Kyoto University, located in Takatsuki, Japan (Takatsuki Farm, 34°51’N, 135°37’E) in the spring of 2015. To conduct fine mapping of *Pat-k*, we developed an F_4_ population (*n* = 507) from three F_3_ plants that were heterozygous at the *Pat-k* locus. Seeds of the F_4_ population were sown in September 2015, and each F_4_ seedling was subjected to recombinant screening to seek recombinants in the corresponding area of the *Pat-k* locus. Selected F_4_ recombinants were grown in the greenhouse of the Takatsuki Farm in the autumn of 2015.

For expression analysis, we used two tomato cultivars, a non-parthenocarpic tomato cultivar, ‘Louis 60’ (TAKII SEED, Kyoto, Japan) and ‘MPK-1’. The plants of the two cultivars were grown in a greenhouse at the Takatsuki Farm in the autumn of 2015 for sampling ovaries and at the Kizu Experimental Farm of Kyoto University at Kizugawa, Japan (Kizu Farm, 34°73′N, 135°84′E) in the spring of 2016 for sampling buds.

For histological analysis of ovules, an F_3_ population was developed from two F_2_ plants that were heterozygous for the *Pat-k* locus. Seeds of the F_3_ population were sown in April 2017. We grew them in the greenhouse at the Kizu Farm in the spring of 2017. In addition, ‘Micro-Tom’ and ‘MPK-1’ were grown in a greenhouse at the Takatsuki Farm in the autumn of 2013.

### The evaluation of parthenocarpy and seed production

We obtained phenotype data for the F_2_, F_3_, and F_4_ populations using the following procedure. We emasculated five flowers within one or two flower clusters at one day before anthesis (− 1 DAA). Ten flowers within more than two flower clusters were pollinated at anthesis (0 DAA). After weighing the emasculated and pollinated fruits, we checked for the absence of seeds in the emasculated fruits and counted the number of seeds in the pollinated fruits. Parthenocarpy level (PL) (i.e., percentage of average weight of emasculated fruits to that of pollinated fruits) was used to evaluate the degree of parthenocarpy. PL of any plant with emasculated ovaries that dropped (or did not grow) was defined as 0%. All PL data were arcsine transformed prior to QTL analysis in order to improve the normality of the distribution.

### DNA extraction

We used frozen, young leaves for extracting DNA. The DNA for our single nucleotide polymorphism (SNP) analysis, and for whole genome re-sequencing of ‘MPK-1’, was extracted using a DNeasy Plant Mini Kit (Qiagen GmbH, Hilden, Germany). For the other investigations, we extracted DNA with a Nucleon PhytoPure kit (GE Healthcare, Buckinghamshire, UK), according to the manufacturer’s instructions.

### SNP analysis and linkage map construction

We conducted SNP analysis to detect SNP genotypes of the 89 plants of the F_2_ population by using the Axiom® tomato Genotyping Array (Takara Bio, Shiga, Japan), which contains 52,425 SNPs [15]. We selected SNPs that satisfied following conditions for construction of a linkage map: (1) homozygous in ‘Micro-Tom’ and ‘MPK-1’, (2) polymorphic between ‘Micro-Tom’ and ‘MPK-1’, and (3) less than 10% missing data. The linkage groups were formed according to the information of the location of the SNPs in the tomato genome (SL2.40). A genetic map was constructed using JoinMap4.1 mapping software with default settings of a maximum-likelihood mapping algorithm.

### QTL analysis

QTL analyses were performed with the linkage map and phenotype data from the F_2_ population: arcsine transformed PL and number of seeds. Composite interval mapping was performed with Windows QTL Cartographer software v.2.5 [16]. The logarithm of odds (LOD) thresholds of PL and number of seeds were determined by one thousand permutation tests at the 5% level for each trait. In order to evaluate the effect of the detected QTLs, we classified the F_2_ population according to the genotypes of the nearest markers linked to the QTLs. Multiple comparisons were conducted with the Tukey–Kramer test.

### Whole genome re-sequencing of ‘MPK-1’

Whole genome re-sequencing of ‘MPK-1’ was performed by the Macrogen Japan service (Macrogen Japan, Tokyo, Japan). The 100 bp pair-end reads were generated using the HiSeq2000 system (Illumina, California, USA). The quality of these data was evaluated based on total reads and the content of GC, AT, Q20, and Q30 (Additional file 1: Table S1). The obtained data were aligned to the tomato reference genome sequence (SL2.40) using BWA software in the DDBJ Read Annotation Pipeline [17]. SNPs between the tomato reference genome (‘Heinz 1706’) and ‘MPK-1’ were identified with SAMtools software. Integrative Genomics Viewer (IGV) free software was used to view the sequences [18].

### Fine mapping of *Pat-k*

We selected F_4_ recombinants using two DNA markers, Affx-93,173,536 and TGS0486, which flanked the *Pat-k* locus in this study. Affx-93,173,536 was a cleaved amplified polymorphic sequence (CAPS) marker, which was designed using SNP data between ‘Micro-Tom’ and ‘MPK-1’ (Additional file 1: Table S2). TGS0486 was an SSR marker, which was linked to *Pat-k* [1]. In addition, we acquired SNPs between ‘Micro-Tom’ and ‘MPK-1’ by comparing two SNP data sets: (1) SNPs between ‘Heinz 1706’ and ‘Micro-Tom’ genome from TOMATOMICS [19] and (2) SNPs between ‘Heinz 1706’ and the ‘MPK-1’ genome obtained in this study. We selected four SNPs to increase the map resolution near the *Pat-k* locus. We also developed CAPS markers to detect genotypes of the F_4_ recombinants for two SNPs (SNP6 and SNP13). For the other two SNPs (SNP17 and SNP19), we applied the direct sequencing method (Additional file 1: Table S2).

### Genomic sequence analysis of *Solyc01g093960* in ‘MPK-1’

We determined the genome sequence corresponding to the open reading frame of *Solyc01g093960* in the International Tomato Annotation Group, release 2.40, using whole genome re-sequencing data of ‘MPK-1’. An insertion sequence, which was found at the first intron of *Solyc01g093960*, was amplified using a set of primers: forward primer sequence (093960_fwd) (5′-ACAGTTGATGTGTGCCTTTGTCTCTCAACAA-3′) and reverse primer sequence (093960_rev) (5’-GAGAGAGTGAAAGACAGTGAGGTCA-3′). PCR reactions were performed in a total volume of 50 μL PrimeSTAR® GXL DNA Polymerase (Takara Bio, shiga, Japan), according to the manufacturer’s instructions. Amplification was performed under the following conditions: 94 °C for 5 min, 35 cycles of 98 °C for 10 s, and 68 °C for 8 min; a final extension step was conducted at 72 °C for 7 min. The PCR products were run on 1.0% agarose gels and stained with Midori Green Advance (NIPPON Genetics,Tokyo, Japan) to confirm their amplification, after which they were purified with FastGene Gel/PCR Extraction Kit (NIPPON Genetics, Tokyo, Japan). Primer walking of the insertion fragment was performed by the FASMAC (Fasmac, Kanagawa, Japan) and Hokkaido System science (Hokkaido System Science, Hokkaido, Japan) sequencing service using 15 primers (Additional file 1: Table S3).

### Quantitative RT-PCR analysis of *Solyc01g093960*

We collected buds of three sizes (6, 8, and 10 mm) and ovaries at − 1, 0, 1, 3, 5, and 7 days after anthesis (DAA). Both unpollinated and pollinated ovaries were prepared by emasculation at − 1 DAA and pollination at 0 DAA, respectively. The collected buds and ovaries were frozen in liquid N_2_ and stored at − 80 °C in a freezer until ready to extract RNA. Total RNA was extracted using Sepasol®-RNA I Super G (NACALAI TESQUE, Kyoto, Japan), according to the manufacturer’s instructions. cDNA was synthesized using ReverTra Ace qPCR RT Kit (Toyobo, Tokyo, Japan). Quantitative RT-PCR was performed with the LightCycler® 480 System (Roche Applied Science, Mannheim, Germany) using THUNDERBIRD® SYBR qPCR Mix (Toyobo, Tokyo, Japan). The primers used for expression analysis of *Solyc01g093960* were designed using Primer 3 plus [20] (Additional file 1: Table S4). Transcript levels for the genes were normalized with the expression of the housekeeping gene, *Sl-Actin* [21] (Additional file 1: Table S4). Amplification was performed under the following conditions: 95 °C for 1 min, 45 cycles of 95 °C for 10 s, 60 °C for 25 s, and 72 °C for 25 s; a final step at 40 °C for 30 s completed the amplification procedure. Three biological replicates were performed.

### The observation of ovules with light microscopy

At 0 DAA, two ovaries were sampled in each of the three F_3_ plants that were homozygous for the ‘MPK-1’ allele and the three F_3_ plants that were homozygous for the ‘Micro-Tom’ allele at the *Pat-k* locus. In all the F_3_ plants we used, the genotype at the nearest marker of two QTLs for number of seeds on chromosome 2 and 4 were ‘MPK-1’ homozygous.

Ovaries were fixed with formalin-acetic acid-alcohol: 50% ethanol, glacial acetic acid, and formalin at the ratio of 18:1:1 (*v*/v/v). After one night, the ovaries were washed with running tap water and then dehydrated with a series of ethanol treatments: 30%, 70%, 90%, 99%, and 100% ethanol (*v*/v). After substituting ethanol with Technovit 7100 resin (Heraeus Kulzer, Wehrheim, Germany), the samples were embedded in resin and coagulated in a mold. Transverse sections (2 μm thick) were prepared with a rotary microtome and then stained with toluidine blue O to observe the morphology of ovules with light microscopy. Based on this observation, ovules were separated into the following two types: normal ovule (with micropyle that was properly closed) and abnormal ovule (with micropyle that had a cavity). We observed 10–12 ovules in each plant and evaluated ovule aberrancy as the percentage of the number of abnormal ovules to all observed ovules.

## Results

### Distribution of the PL and number of seeds

**Fig. 1:**
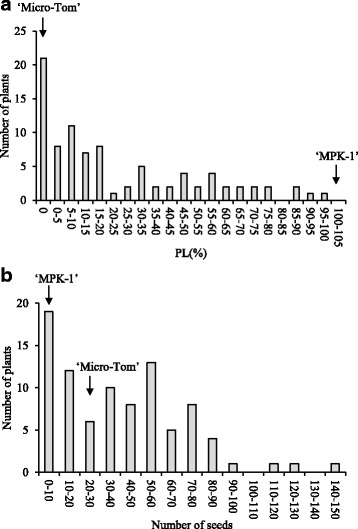
Frequency distribution of PL and number of seeds in the F_2_ population (*n* = 89). **a** PL, **b** number of seeds

The PL of ‘Micro-Tom’ was 0% because its emasculated ovaries did not grow after anthesis. In contrast, emasculated ovaries of ‘MPK-1’ grew to the almost the same size as pollinated fruits (its PL was 104%). The mean number of seeds produced by ‘Micro-Tom’ was 28.9, whereas ‘MPK-1’ produced no seeds. The F_2_ population showed a continuous distribution in PL values (ranging from 0% to 98.2%), whereas mean number of seeds ranged from 0 to 141.5 (Fig. 1).

### Linkage map construction

Of the 52,425 SNPs in the genotyping array, 4238 SNPs fulfilled our requirements. The redundant markers detecting no recombination in the F_2_ population were removed because they could not provide any additional information. The selected 1431 SNPs were applied to the construction of our linkage map (Additional file 1: Table S5). The linkage map covered a total of 2082 cM and contained 12 chromosomes. The average marker interval of this map was 1.5 cM.

### QTL analysis for PL

**Fig. 2:**
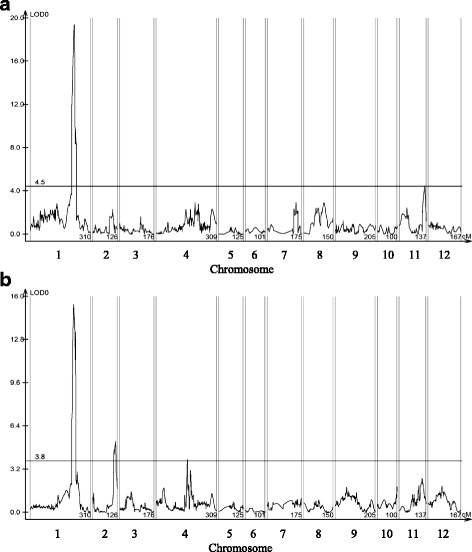
Lod score distribution in the QTL analyses in the F_2_ population of ‘Micro-Tom’ and ‘MPK-1’. **a** QTLs of PL after arcsine transformation, **b** QTLs of number of seeds

In the QTL analysis for PL, we detected only one QTL, *qpat1.1* at 224.9 cM of chromosome 1 (LOD = 19.4, R^2^ = 70.8) (Fig. 2a, Table 1). The additive effects of *qpat1.1* were positive, indicating that the allele of ‘MPK-1’ increased PL. Mean PL was 12.5% for plants homozygous for the ‘Micro-Tom’ allele at the nearest marker to *qpat1.1* (Affx-93,180,219), whereas the PL was 55.7% for plants homozygous for the ‘MPK-1’ allele (Table 2). There was a significant difference between them at the 5% level.

**Table 1:** QTLs for PL after arcsine transformation and number of seeds detected in the F_2_ population

Trait	Chromosome	Region of QTL	Nearest marker	Position	LOD	Additive effect	Dominant effect	R^2e^
PL	Chr1	Affx-93,162,137-Affx-93,170,983	Affx-93,180,219	224.9	19.4	0.4	−0.3	70.8
Number of seeds	Chr1	Affx-93,162,137-Affx-107,980,374	Affx-93,153,041	223.2	15.4	−32.3	19.2	61.9
	Chr2	Affx-108,001,187-Affx-107,991,951	Affx-108,004,902	115.7	5.3	8.1	11.9	3.4
	Chr4	Affx-107,974,589	Affx-107,974,589	157.9	3.9	31.1	−0.1	9.8

**Table 2:** Average PL of each genotypes for *qpat1.1* in the F_2_ and F_3_ populations

Population	*qpat1.1* (Affx-93,180,219)	PL (%)			*n* ^d^
		mean	SE^c^		
F_2_	M^b^	12.5	3.4	a^a^	25
	H	11.3	2.1	a	39
	K	55.7	5.2	b	25
F_3_	M	15.5	8.8	a	13
	H	17.3	11.2	a	20
	K	91.2	17.4	b	9

^a^Values followed by the same letter are not significantly different at *P* < 0.05 by the Tukey–Kramer test

^b^Genotypes of the nearest marker. M = ‘Micro-Tom’ homozygous, H = Heterozygous, K = ‘MPK-1’ homozygous

^c^SE: standard error

^d^n: number of plants for each genotype

**Fig. 3:**
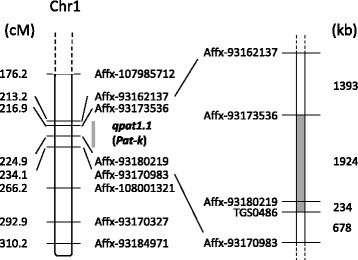
Genetic and physical map of the chromosome region at the *Pat-k* locus. Genetic map of the *Pat-k* locus (left side). Position of the markers are indicated in cM. The gray bar represents the position of the *qpat1.1* (*Pat-k*) locus suggested by QTL analysis in this experiment. Physical map of the *Pat-k* locus (right side), which was delimited to the region between Affx-93,173,536 and TGS0486 using the data of the F_3_ population. The gray region represents the *Pat-k* locus, which was narrowed. Distance of the markers are indicated in kb

To confirm the effect of *qpat1.1*, we developed an F_3_ population from a single F_2_ plant with a heterozygous allele for Affx-93,180,219. The mean PL of plants homozygous for the ‘MPK-1’ allele at the Affx-93,180,219 was significantly higher than that of plants homozygous for the ‘Micro-Tom’ allele in the F_3_ population (Table 2). Takisawa [1] reported that parthenocarpy in ‘MPK-1’ is controlled by the parthenocarpic gene, *Pat-k,* which is linked to the SSR marker, TGS0486 (positioned at 77847211–77847468 on chromosome 1 in SL2.40). The physical distance between TGS0486 and Affx-93,180,219 was only 234 kb (Fig. 3b). This result strongly suggested that *qpat1.1* is identical to *Pat-k*. Therefore, we hereafter refer to *qpat1.1* as *Pat-k*. In addition, we genotyped two markers (Affx-93,173,536 and TGS0486) in nine F_3_ plants that had the high PL scores and were homozygous for the ‘MPK-1’ allele at the Affx-93,180,219. As a result, we found that three of the nine plants were heterozygous at Affx-93173536 and homozygous for the ‘MPK-1’ allele at TGS0486. In addition, one plant was heterozygous at TGS0486 and homozygous for the ‘MPK-1’ allele at Affx-93173536. Therefore, we could narrow the chromosome region assumed for the *Pat-k* locus to be located between Affx-93,173,536 and TGS0486 (Fig. 3b).

### QTL analysis for seed production

Three QTLs for number of seeds were detected on chromosomes 1, 2, and 4, which were designated as *qsn1.1*, *qsn2.1*, and *qsn4.1*, respectively (Fig. 2b, Table 1). The additive effects of *qsn1.1* were negative, whereas those of *qsn2.1* and *qsn4.1* were positive, indicating that the allele for reducing seed production was the ‘MPK-1’ allele on *qsn1.1* and the ‘Micro-Tom’ allele on *qsn2.1* and *qsn4.1*. The QTL, *qsn1.1* was detected at 223.2 cM of chromosome 1 (LOD 15.4, R^2^ = 61.9), whereas *qsn2.1* was detected at 115.7 cM of chromosome 2 (LOD 5.3, R^2^ = 3.4) and *qsn4.1* was detected at 157.9 cM of chromosome 4 (LOD 3.9, R^2^ = 9.8) (Table 1). F_2_ plants homozygous for the ‘MPK-1’ allele at the nearest marker to *qsn1.1* (Affx-93,153,041) had significantly fewer seeds than plants homozygous for the ‘Micro-Tom’ allele (Table 3). In contrast, plants homozygous for the ‘Micro-Tom’ allele at the nearest marker to *qsn2.1* (Affx-108,004,902) and *qsn4.1* (Affx-107,974,589) had fewer seeds than plants homozygous for the ‘MPK-1’ allele; however, they were not statistically significant.

**Table 3:** Average number of seeds of each genotype for *qsn1.1*, *qsn2.1*, and *qsn4.1* in the F_2_ population

Locus	Genotype	seed number			n^d^
		mean	SE^c^		
*qsn1.1*	M^b^	55.1	4.1	a^a^	27
	H	52.9	4.9	a	36
	K	8.2	1.4	b	26
*qsn2.1*	M	30.3	4.1	a	24
	H	44.5	4.8	a	42
	K	43.7	7.7	a	23
*qsn4.1*	M	34.2	5.3	a	19
	H	34.9	4.4	a	40
	K	52.3	8.0	a	23

^a^Values followed by the same letter are not significantly different at *P* < 0.05 using the Tukey–Kramer test

^b^Genotypes of the nearest marker. M = ‘Micro-Tom’ homozygous, H = Heterozygous, K = ‘MPK-1’ homozygous

^c^SE: standard error

^d^n: number of plants for each genotype

### Whole genome re-sequencing and fine mapping of *Pat-k*

We performed whole genome re-sequencing of ‘MPK-1’ to isolate *Pat-k*. We obtained information on SNPs between ‘Heinz 1706’ and ‘MPK-1’ using our re-sequencing data. In addition, SNP data between ‘Heinz 1706’ and ‘Micro-Tom’ genome were obtained from TOMATOMICS. By comparing these two data sources, we acquired the polymorphic SNPs between ‘Micro-Tom’ and ‘MPK-1’. We found 58 SNPs in the region between Affx-93,173,536 and TGS0486 between ‘Micro-Tom’ and ‘MPK-1’ (Additional file 1: Table S6). Using these SNP data, we developed two CAPS markers (SNP17 and SNP19). We then performed fine mapping with the F_4_ population, which was from three F_3_ plants heterozygous for the two markers, Affx-93,173,536 and TGS0486 to narrow the interval of the *Pat-k* locus. Of the 507 plants, 43 plants displayed recombinations for the region between Affx-93,173,536 and TGS0486. They were genotyped with two CAPS markers (SNP17 and SNP19) and two SNP markers (SNP6 and SNP13) in the region between Affx-93,173,536 and TGS0486. We then scored these 43 plants for their degree of PL and number of seeds.

**Fig. 4:**
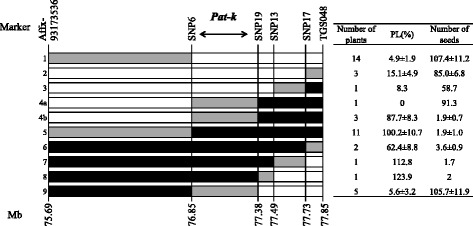
Fine mapping of *Pat-k*. Black block stands for homozygous ‘MPK-1’, white block stands for heterozygous, and gray block stands for interval where crossover took place. Numbers on left side of figure shows classification numbers by the genotype of the corresponding area. Numbers under the bars indicated the position of the above markers on chromosome 1 in Mb (SL2.40). *Pat-k* was located in the 529 kb region between SNP6 and SNP19

We could divide recombinant plants into nine groups by their genotypes (Fig. 4). Of the nine groups, only Group 4 included both low-scoring PL plants and high-scoring PL plants. Consequently, we concluded that *Pat-k* located in the region between SNP6 and SNP19 with a physical distance of 529 kb. In addition, the number of seeds of these groups closely corresponded to PL values. The groups in which PL was > 62.4% (Group 4b and 5–8 in Fig. 4) produced fewer seeds on average (< 3.6 per plant), whereas the other group, in which PL was < 15.1% (Group 1–3, 4a, and 9 in Fig. 4), produced more seeds (mean > 58.7) (Fig. 4).

### Map-based cloning of *Pat-k*

**Fig. 5:**
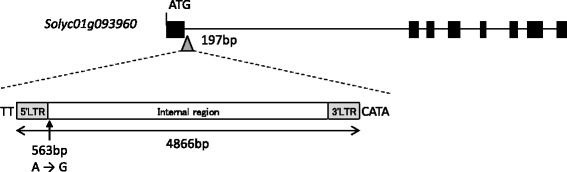
The structure of *Solyc01g093960* of ‘Heinz 1706’ and the insertion fragment in ‘MPK-1’. Black box and black line stand for the exons and introns of *Solyc01g093960*, respectively. The gray triangle indicates the insertion site of CopiaSL_37 retrotransposon. The 2 bp sequence (TT) and 4 bp sequence (CATA) were added to the left and right insertion site, respectively. Arrow indicates a single base substitution from A to G

Based on the Sol Genomics Network database [22], there are 60 genes within the 529 kb region between SNP6 and SNP19. By comparing the sequence of ‘MPK-1’ and ‘Heinz 1706’ in this region (using IGV), we could predict that a large DNA fragment was located in a region of *Solyc01g093960*. By performing PCR analysis, we found an approximate 5 kb insertion in this region of ‘MPK-1’ (Additional file 1: Fig. S1). By sequencing the insertion region using primer walking procedures, we identified a 4872 bp insertion at 197 bp downstream of the start codon of *Solyc01g093960*. The inserted sequence included a 4866 bp segment that was homologous to the LTR retrotransposon CopiaSL_37, which had one SNP at 563 bp downstream of the 5’LTR (Fig. 5). In addition, after comparing the sequence data between ‘MPK-1’ and ‘Heinz 1706’ (by IGV and direct sequencing method), we found that there were no polymorphisms in the exon of all 60 genes predicted in the 529 kb region.

### The expression analysis of *Solyc01g093960* in ovaries

**Fig. 6:**
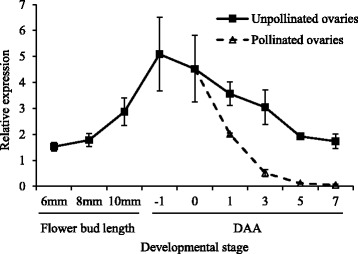
Transcript levels of *Solyc01g093960* in the buds and ovaries of ‘Louis 60’. The unpollinated ovaries and pollinated ovaries were prepared by emasculation at − 1 DAA and pollination at 0 DAA. We examined the transcript levels of *Solyc01g093960* in the buds of the size 6, 8, and 10 mm and ovaries at − 1, 0, 1, 3, 5, and 7 DAA. Values indicate means ± SE (*n* = 3)

**Fig. 7:**
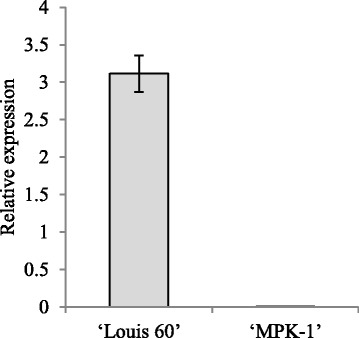
Transcript levels of *Solyc01g093960* in the ‘Louis 60’ and ‘MPK-1’ ovaries at − 1 DAA. Values indicate means ± SE (*n* = 3)

Our expression analysis showed that transcript levels of *Solyc01g093960* were highest at − 1 DAA in the ovaries of ‘Louis 60’, a non-parthenocarpic tomato cultivar (Fig. 6). The expression level declined considerably in the pollinated ovaries of ‘Louis 60’ after pollination. On the other hand, the transcript level of *Solyc01g093960* was mostly undetectable in the ovary of ‘MPK-1’ at − 1 DAA (Fig. 7).

### The relationship between *Pat-k* and the abnormal morphology of ovules

**Fig. 8:**
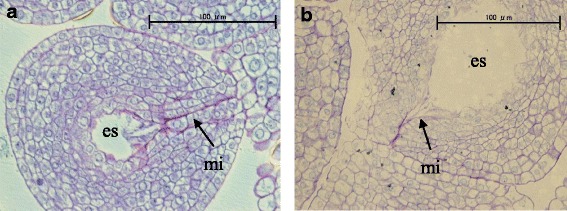
Morphology of ovules at anthesis. **a** The ovule of ‘Micro-Tom’ had a normal micropyle that was tightly closed and looked like a purple line. **b** the ovule of ‘MPK-1’ had an abnormal micropyle that had a cavity. es = embryo sac, mi = micropyle

To clarify the relationship between *Pat-k* and ovule structure, we observed ovules in ‘Micro-Tom’, ‘MPK-1’, and the F_3_ population that was homozygous for the ‘Micro-Tom’ or ‘MPK-1’ allele at the *Pat-k* locus. We found abnormal ovules in ‘MPK-1’ and normal ovules in ‘Micro-Tom’ (Fig. 8). The normal ovules had micropyles that closed properly. In contrast, the abnormal ovules had micropyles with a cavity. There were no abnormal ovules in the ‘Micro-Tom’, whereas 40% were abnormal in ‘MPK-1’. There were no abnormal ovules in the F_3_ progeny of ‘Micro-Tom’ that were homozygous at the *Pat-k* locus, whereas 51% of ovules were abnormal in the F_3_ progeny of ‘MPK-1’ that were homozygous at the *Pat-k* locus.

## Discussion

### QTL for parthenocarpy in ‘MPK-1’

In this study, we first performed QTL analysis to confirm the factors related to parthenocarpy in ‘MPK-1’. Then, we identified only one major QTL, *qpat1.1* for PL on chromosome 1. We had previously reported that parthenocarpy in ‘MPK-1’ is controlled by a parthenocarpic gene, *Pat-k*, which is semi-dominant and located on chromosome 1 [1]. We considered *qpat1.1* to be *Pat-k* because they were both detected in the same region of the chromosome 1. However, *qpat1.1* appears to be recessive, although we had previously reported that *Pat-k* was semi-dominant. This discrepancy might be due to the difference in the way we evaluated the parthenocarpy phenotype in the two studies. In the previous report, we evaluated the parthenocarpic phenotype based on the initial development of emasculated ovaries, and separated the phenotypes into three groups: no parthenocarpy (setting fruit, but not growing fruit or dropping flowers), weak parthenocarpy (starting to grow fruit after anthesis), and strong parthenocarpy (starting to grow fruit before or at anthesis) [1]. In contrast, in this study, we evaluated the parthenocarpic phenotype by quantitatively examining the mean final weight of parthenocarpic fruits to that of pollinated fruits. Plants that were heterozygous at the *Pat-k* locus may promote the initial development of emasculated ovaries to some extent, although the ovaries do not grow as large as pollinated fruits. This suggests that the heterozygous allele on the *Pat-k* locus affects the initial development of emasculated ovaries, rather than final size of parthenocarpic fruit, which may cause the difference in the effect of genes.

### The retrotransposon insertion in *SlAGL6* results in parthenocarpy of ‘MPK-1’

In a previous study, *Pat-k* was mapped at a position close to the SSR marker TGS0486 in chromosome 1 [1]. We conducted fine mapping and map-based cloning to isolate *Pat-k*. Consequently, we found that the 4866 bp LTR retrotransposon CopiaSL_37 was inserted at the first intron of *Solyc01g093960* in the relevant region of chromosome 1. In addition, there was no polymorphism in the exon of all 60 genes in the region delimited by fine mapping. These results suggest that *Pat-k* is identical to *Solyc01g093960*. Our expression analysis showed that the transcript level of *Solyc01g093960* is highest in ovaries of ‘Louis 60’ whose PL was 0% at − 1 DAA. In addition, whereas the transcript levels of *Solyc01g093960* in ‘Louis 60’ and ‘Micro-Tom’ is 3.11 and 0.79 (data not shown), respectively at − 1 DAA, it is mostly undetectable in ‘MPK-1’ ovaries at that time. Yao [23] reported that the retrotransposon insertion in intron 4 and intron 6 of the MADS-box transcription factor (*MdPI*) abolished its gene expression, resulting in seedless phenotypes. The source for the high locule number phenotype is a down-regulation of the *fasciated* gene, which encodes a YABBY-like transcription factor, by a large insertion in the first intron [24]. Considering these reports, down-regulation of *Solyc01g093960* in ‘MPK-1’ might also be caused by a retrotransposon insertion in an intron. Very recently, Klap [25] reported that a mutation in *Solyc01g093960*, which encodes *SlAGAMOUS-LIKE 6* (*SlAGL6*), was responsible for the parthenocarpic phenotype in tomato. In addition, silencing *SlAGL6* led to abnormally-fused sepals and light green petals that were smaller-than-normal [26]. Fused sepals were also observed in ‘MPK-1’ [1]. Taken together, we conclude that the parthenocarpic phenotype of ‘MPK-1’ is caused by down-regulation of *SlAGL6* through a retrotransposon insertion and that the allele of ‘MPK-1’ in the *Pat-k* locus originated from a natural mutation.

Hosokawa [7] reported that ‘MPK-1’ was derived from a cross between a non-parthenocarpic cultivar and a variant from a self-fertilized descendant of ‘Severianin’ with the parthenocarpic gene, *pat-2*. Therefore, ‘MPK-1’ was considered to have *pat-2*, but in reality, it has *Pat-k*/*SlAGL6* instead [1, 8]. The parthenocarpic parent of ‘MPK-1’ has been lost. Therefore, the origin of *Pat-k*/*SlAGL6* remains a mystery. However, in this study, we showed that the parthenocarpic phenotype of ‘MPK-1’ is due to the insertion of the retrotransposon, CopiaSL_37. The CopiaSL_37 is reported to be potentially active and autonomous [27], which suggests that the insertion event of CopiaSL_37 may have happened in the parthenocarpic parent of ‘MPK-1’ or during the selection process, and then ‘MPK-1’ succeeded it.

### QTL for number of seeds in ‘MPK-1’

We identified three QTLs for number of seeds. The QTL, *qsn1.1* was located very close to *qpat1.1*. In addition, PL score and numbers of seeds produced were completely co-segregated in the F_4_ recombinants. These results suggest that *qsn1.1* is identical to *Pat-k*/*SlAGL6*. Klap [25] reported that plants that are homozygous for the mutated allele of *SlAGL6* set mostly parthenocarpic fruits (and a few seeded fruits), which is consistent with our results. Therefore, we conclude that the inhibition of seed formation in ‘MPK-1’ was caused by down-regulation of *Pat-k*/*SlAGL6*.

Other QTLs for seed production, *qsn2.1* and *qsn4.1*, showed positive additive effects, indicating that an allele from ‘Micro-Tom’ was responsible for the low number of seeds. ‘Micro-Tom’, a dwarf cultivar, is regarded as a model cultivar of tomato. The dwarf phenotype of ‘Micro-Tom’ results from mutations in three major recessive loci: *DWARF* gene (*Solyc02g089160*), *SELFPRUNING* gene (*Solyc06g074350*), and *SlGLK2* gene [28–30]. Of the three genes, *DWARF* is located within the region of the *qsn2.1* locus. The *DWARF* gene encodes a cytochrome P450 protein, which functions in the brassinosteroid biosynthesis pathway. Jiang [31] reported that brassinosteroid-deficient and -insensitive mutants have fewer ovules or seeds than the wild type in *Arabidopsis*. These results suggest that *qsn2.1* may be *dwarf* and it reduces the number of seeds through the inhibition of brassinosteroid biosynthesis. It is to be noted that seed production is inhibited by *dwarf* in ‘Micro-Tom’.

We grouped F_2_ progeny according to the genotypes of *qsn4.1* and then compared the average weight of pollinated fruit among genotypes. Our results show that the average weight of pollinated fruit is less in plants with the ‘Micro-Tom’ homozygous allele than in plants with the heterozygous allele and the ‘MPK-1’ homozygous allele. Actually, the QTL for pollinated fruit weight was detected on chromosome 4 in the F_2_ population (data not shown). These results suggest that *qsn4.1* is responsible for reduced fruit size, which also causes a reduction in the number of seeds.

### The relationship between *Pat-k*/*SlAGL6* and abnormal ovule

In eudicots, flowers consist of sepals, petals, stamens, and pistils. Based on studies in two eudicot plants, *Arabidopsis thaliana* and *Antirrhinum majus*, an ABC model was established that explains how three classes of genes jointly specify floral organ identity [32]: A class, A and B class, A and C class, and C class determine sepals, petals, stamens, and carpels, respectively. Later, the model was expanded by finding genes of D and E class. D class specifies the ovule [33, 34], whereas E class genes determine the identity of all flower organs and regulates floral meristem determinacy [35–37]. The *AGL6* subfamily of genes belongs to E class, and they function in flower development, such as the *SEPALLATA* genes, which are well characterized E class genes [38].

Yu [26] reported that *SlAGL6* is a potential E-function gene because its expression profile was similar to *Petunia AGL6,* lineage member. Pattison [39] reported that the transcript level of *SlAGL6* in the ovules is higher than in the other tissues of the ovary. High expression levels of *AGL6*-like genes in ovules seem to be well conserved among some species [40–43]. Rijpkema [42] suggested that *PhAGL6* plays a role during ovule development, based on its expression pattern. The osmads6 mutant, which is a mutation of *OsMADS6* (*AGL6*-like genes), has defective ovules, which leads to fewer seed set [41]. In our study, we observed ovules with abnormal micropyles only in progenies homozygous for the ‘MPK-1’ allele at the *Pat-k*/*SlAGL6* locus, which suggests that *Pat-k*/*SlAGL6* is involved with ovule formation in tomato and that its down-regulation causes abnormal ovules. In addition, the percentage of germination of hybrid seeds between ‘MPK-1’ and ‘Louis 60’ was low and the internal structure of the seeds was abnormal [8]. This suggests that abnormal ovules inhibit normal seed formation. It was reported that *SlAGL6* is a single, recessives gene for parthenocarpy and there are no adverse effects on fruit weight or shape or on vegetative traits in SlAGL6-mutated lines [25]. These traits indicate that *Pat-k*/*SlAGL6* should be a desirable gene for breeding parthenocarpic cultivars. However, in our study, the down-regulation of *Pat-k*/*SlAGL6* not only exhibits stable parthenocarpy, but also inhibits seed formation via abnormal ovule formation. Although the treatment of gibberellin biosynthesis inhibitor or inhibitor of auxin action increases the number of seeds in ‘Renaissance’, a parthenocarpic cultivar with *pat-2*, [44, 45], they have no effect on seed production in ‘MPK-1’ (unpublished data). This result shows that it is difficult to increase the number of seeds in a *SlAGL6*-mutated line using a chemical that affects the biosynthesis or signaling of plant hormones. However, Klap [25] reported that environmental conditions and genetic background together affect seed production, indicating that it is possible to increase the number of seeds by finding good cross combination or by improving field conditions for seed production.

## Conclusion

In this study, we identified one QTL for PL and three QTLs for seed production in tomato. Fine mapping and a whole genome re-sequencing of ‘MPK-1’ identified that *Pat-k* was identical to *SlAGL6*. The insertion of retrotransposon in the first intron of *SlAGL6* down-regulates the transcript level of *SlAGL6* in ‘MPK-1’ ovaries, which causes parthenocarpy and low seed set in ‘MPK-1’. Moreover, all F_3_ progenies homozygous for the ‘MPK-1’ allele of the *Pat-k*/*SlAGL6* locus had ovules with abnormal micropyles, which indicates that *Pat-k*/*SlAGL6* is involved with ovule formation and that its down-regulation causes abnormal ovule formation and low seed set.

## Additional file

Additional file 1 **Table S1.** Raw data status of whole genome re-sequencing of ‘MPK-1’. **Table S2.** Primer sequences used for fine mapping. **Table S3.** Primer sequences used for primer walking of the insertion in the *Solyc01g093960* gene. **Table S4.** Primer sequences used for quantitative RT-PCR analysis of the *Solyc01g093960* gene. **Table S5.** The information of SNPs used in QTL analysis in this study. **Table S6. **The information of SNPs for fine mapping of *Pat-k* in this study. **Figure S1.** Amplification products produced by the primers of 093960_fwd and 093960_rev. (XLSX 136 kb)

## Abbreviations

CAPS: Cleaved amplified polymorphic sequences
DAA: Days after anthesis
IGV: Integrative genomics viewer
LOD: Logarithm of odds
PL: Parthenocarpy level
QTL: Quantitative trait locus
SlAGL6: SlAGAMOUS-LIKE 6
SNP: Single nucleotide polymorphism
